# Molecular mechanisms of inhibiting glucosyltransferases for biofilm formation in *Streptococcus mutans*

**DOI:** 10.1038/s41368-021-00137-1

**Published:** 2021-09-30

**Authors:** Qiong Zhang, Qizhao Ma, Yan Wang, Hui Wu, Jing Zou

**Affiliations:** 1 State Key Laboratory of Oral Diseases, National Clinical Research Center for Oral Diseases and Department of Pediatric Dentistry, West China Hospital of Stomatology, Sichuan University, Chengdu, China; 2 Department of Integrative Biomedical and Diagnostic Sciences, Oregon Health and Science University School of Dentistry, Portland, OR USA

**Keywords:** Biofilms, Bacteria

## Abstract

Glucosyltransferases (Gtfs) play critical roles in the etiology and pathogenesis of *Streptococcus mutans (S. mutans)-* mediated dental caries including early childhood caries. Gtfs enhance the biofilm formation and promotes colonization of cariogenic bacteria by generating biofilm extracellular polysaccharides (EPSs), the key virulence property in the cariogenic process. Therefore, Gtfs have become an appealing target for effective therapeutic interventions that inhibit cariogenic biofilms. Importantly, targeting Gtfs selectively impairs the *S. mutans* virulence without affecting *S. mutans* existence or the existence of other species in the oral cavity. Over the past decade, numerous Gtfs inhibitory molecules have been identified, mainly including natural and synthetic compounds and their derivatives, antibodies, and metal ions. These therapeutic agents exert their inhibitory role in inhibiting the expression *gtf* genes and the activities and secretion of Gtfs enzymes with a wide range of sensitivity and effectiveness. Understanding molecular mechanisms of inhibiting Gtfs will contribute to instructing drug combination strategies, which is more effective for inhibiting Gtfs than one drug or class of drugs. This review highlights our current understanding of Gtfs activities and their potential utility, and discusses challenges and opportunities for future exploration of Gtfs as a therapeutic target.

## Introduction

Dental caries is a multifactorial, sugar-dependent disease driven by the dynamic interactions of cariogenic and commensal microbes within dental plaque (biofilms) formed on tooth surfaces.^1,2^ Although dental biofilms are complex consisting of diverse microbial communities, *Streptococcus mutans* (*S. mutans*) has been considered to be the major pathogen in the initiation and development of dental caries, especially in the early childhood caries (ECC).^3^ *S. mutans* possesses several virulence factors, one of which is the sucrose-dependent adhesion mechanism responsible for dental surface colonization. This adhesion mode contributes significantly to the formation of cariogenic biofilms and cariogenicity.^4^ Sucrose-dependent adhesion is mediated by the activities of glucosyltransferases (Gtfs), a family of enzymes that can split sucrose, the only substrate for the Gtfs, into glucose and fructose, and links the glucose moiety together via glycosidic bonds to form a growing polymer of glucan, termed extracellular polysaccharides (EPSs). The EPS in biofilms provide the microorganisms with a unique microenvironment for their growth, metabolism, and survival, and enables microorganisms to become more resistant to harsh and challenging environmental conditions, host immunity, and traditional antimicrobial therapies, as well as prevents dissociation and enhances its mechanical stability.^5^ More importantly, the interactions between *S. mutans* and certain members of the dental plaque community through Gtfs have recently been shown to exert a major influence on the development and pathogenicity of dental plaque. For example, *Veillonella parvula*, an oral bacterium that prefers to consume lactic acid as a carbon source, alters *S. mutans* virulence factors increasing the expressions of Gtfs in a dual-species biofilm model, suggesting it is engaged in the development of *S. mutans*-mediated dental caries.^6^ In addition to bacteria, fungi can enhance the *S. mutans* pathogenicity via their impact on the expressions of *S. mutans* Gtfs. It has been demonstrated that the cross-kingdom interactions between *Candida albicans* and *S. mutans* depend on Gtfs activity, by which increasing the accumulation of *S. mutans* is associated with the development of ECC.^7–10^

*S. mutans* produces three Gtfs enzymes (GtfB, GtfC, and GtfD), encoded by the *gtfB*, *gtfC*, and *gtfD* genes, respectively, of which the expression is distinct but related. *gtfB* (4.4 kb) and *gtfC* (4.3 kb) are in an operon arrangement separated by 198 bp, whose promoters appear to be coordinately regulated, suggesting they can be co-transcribed and are subjected to the similar regulatory mechanism, whereas *gtfD* (5.3 kb) is located upstream of the *gtfB/C* locus, which has an independent promoter and is not linked to the *gtfB/C* locus.^11,12^ A similar structure is found in all Gtfs, in which GtfB and GtfC are highly homologous sharing ~75% of amino acid sequences, and GtfD possesses 50% sequence identity to GtfB and GtfC. All Gtfs have three distinct functional domains: the N-terminal variable junction domain, the highly conserved catalytic domain, and the C-terminal glucan-binding (GB) domain.^13,14^ The activities of Gtfs are mediated through both catalytic and GB functions.^15,16^ Further, Gtfs have a signal peptide comprising about 38 amino acids at the N terminus, adjacent to which there are about 200 amino acids, a variable domain, which may elucidate the different catalytic functions and binding capacities to different substrates.^17^ Each Gtf plays different and overlapping roles in the formation of dental biofilms. GtfB (formerly known as GtfI) synthesizes mostly insoluble glucans containing α-1,3-linked glucose, GtfC (GtfSI) produces a mixture of insoluble and soluble glucans (α-1,6-linked glucose), and GtfD (GtfS) forms predominantly soluble glucans.^18^ Insoluble glucans facilitate bacterial adherence and accumulation on tooth surfaces, and cause biochemical and structural changes in the matrix of the biofilms, which gives rise to the organism’s resistance to normal mechanical forces of clearance and affords protection from host immune and non-immune defenses; and soluble glucans may be digested and used as a reserve source of energy when exogenous fermentable carbohydrates are exhausted in the oral cavity, which contributes in part to the low pH values observed in cariogenic plaques.^18,19^ Furthermore, binding GtfB to bacteria promotes cell clustering and microbial cohesion within plaque biofilms. GtfC exhibits the greatest affinity for saliva-coated hydroxyapatite (sHA) and displays more binding sites than GtfB and GtfD, whereas GtfD displays relatively fewer binding sites and forms soluble glucans, acting as a primer of GtfB.^20^ Therefore, targeting Gtfs through inhibiting their activity and consequently preventing the synthesis of EPS would impair the *S. mutans* virulence without threatening *S. mutans* existence, or the existence of other species in the oral cavity, which is an appealing strategy to treat dental caries when compared to traditional bactericidal treatments. This precision targeting is advantageous in the inhibition of the formation of cariogenic biofilms and bacterial pathogenesis without promoting bacterial antibiotic resistance, while preserving natural bacterial flora of the mouth.^21,22^ Therapeutic agents against Gtfs exert their inhibitory activities at gene and protein levels through distinct molecular mechanisms. Knowledge of the molecular events underlying the inhibition of Gtfs enzymes facilitates the application of inhibitors alone or in combination with agents against various virulence factors, thus developing more potent and selective therapeutics.

**Fig. 1:**
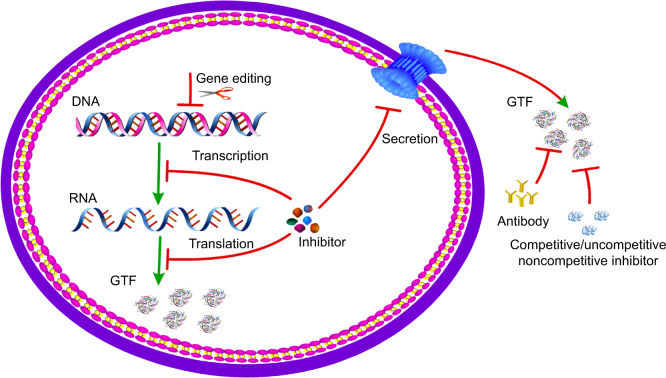
A diagram of the mechanisms of inhibiting glucosyltransferases for biofilm formation. After Gtfs are synthesized inside the bacteria, they are secreted to the surrounding environment where they are functional. Based on research conducted thus far, the molecular mechanism of current inhibitors against Gtfs could be classified into three categories as follows: 1) modulating the expressions of *gtfs* genes at the transcriptional level; 2) reducing the Gtfs enzymatic activities; 3) regulating the secretion of Gtfs outside the cells through dissipating proton motive force across the cell membrane.

Based on research conducted thus far, the molecular mechanism of current inhibitors against the Gtfs activities could be divided into three categories as follows: (1) by modulating the expressions of *gtfs* genes at the transcriptional level; (2) by reducing the Gtfs enzymatic activities; and (3) by inhibiting the secretion of Gtfs outside the cells through dissipating proton motive force across the cell membrane, which is not unique to the secretion of Gtfs, also to other proteins (Fig. 1 and Table 1). This review provides an overview of diverse active mechanisms, by which promising therapeutic agents disrupt pertinent Gtfs activities in *S. mutans* and concurrently negate *S. mutans*-mediated formation of cariogenic biofilms.

**Table 1:** Molecules that inhibit gtfs expressions or Gtfs activities

Category	Therapeutic agents	Culture condition	Concentration and exposure time	Percentage inhibition of *gtfs* expression or Gtfs activities	Reference
Targeting upstream signaling pathways					
*vicR* (TCS)	Cinnamaldehyde	BHI (0.2% sucrose)	250 μg· mL^−1^, 24 h	*gtfB/C* (50%), *gtfD* (90%)	^30^
*vicR* (TCS)	Botulin	THB (1% sucrose)	240 μg· mL^−1^, 24 h	gtfB (87.5%), gtfC (88.4%)	^31^
Mediating the expression of *gtfs* at the transcription level					
	Citrus lemon oil	TPY (1% sucrose)	1.125 mg· mL^−1^, 16 h	*gtfB/D* (95%), *gtfC* (80%)	^41^
	Apigenin	TPY (1% sucrose)	0.1 mmol· L^−1^, 1 h	*gtfB/C* (50%)	^42^
	Deoxynojirimycin	BHI	3 μg· mL^−1^, overnight	*gtfB/C/D* (99.97%)	^44^
Reducing the GTFs enzymatic activities					
	Tris	sHA beads	10 mmol· L^−1^	GtfB (65% in solution, 68% in sHA beads)	^55^
	Green mate	Solution	18 mg· mL^−1^	GtfB/C/D (50%)	^56^
	Roasted mate	Solution	18 mg· mL^−1^	GtfB/C/D (25%)	^56^
	7-epiclusianone	Solution	100 μg· mL^−1^	GtfB (90%), GtfC (80%)	^57,58^
	Cranberry juice	sHA beads	25%, *v*/*v*	GtfB/C (70%–80%), GtfD (40%)	^59,60^
	Polyphenols	Solution	1.0 μmol· L^−1^	GtfB (50%)	^61^
	GTI-0163	Solution	0.05 mmol ·L^−1^	GtfB/C/D (42.4%)	^62^
	Polyherbal mouth rinse	Solution	15% *w*/*v*	GtfB/C/D (95.9%)	^63^
	#43	Solution	25 μmol· L^−1^	GtfB (80%)	^64^
	Zn^2+^	Solution/artificial pellicle	1 mmol· L^−1^	GtfB (58% in solution, 32% in sHA beads), GtfC (25% in solution, 10% in sHA beads), GtfD (50% in solution, 40% in sHA beads)	^83^
	Cu^2+^	Solution/artificial pellicle	5 mmol· L^−1^	GtfB (36% in solution, 46% in sHA beads), GtfC (76% in solution, 32% in sHA beads), GtfD (81% in solution, 69% in sHA beads)	^83^
	Fe^2+^	Solution/artificial pellicle	1 mmol· L^−1^	GtfB (97% in solution, 44% in sHA beads), GtfC (50% in solution, 47% in sHA beads), GtfD (94% in solution, 73% in sHA beads)	^83^
	Fe^3+^	Solution/artificial pellicle	1 mmol· L^−1^	GtfB (72% in solution, 50% in sHA beads), GtfC (29% in solution, 8% in sHA beads), GtfD (67% in solution, 42% in sHA beads)	^83^

## Modulating the expressions of *Gtf* genes

### Targeting upstream signaling pathways

To survive in the continuously changing oral microenvironments, microorganisms use regulatory networks to respond to the stimuli and elicit adaptive responses, which triggers the differential expressions of various regulatory genes. Two-component signal transduction systems (TCSs) are one of the regulatory networks that function as “molecule switches” to modulate the expression of corresponding genes and consequently controlling diverse signaling and metabolic processes including quorum sensing (QS), sporulation, antibiotic/bacteriocin production, and chemotaxis in response to different internal and external stimuli.^23–25^ Typically, TCS works via two regulatory elements consisting of a membrane-associated histidine kinase and a cytoplasmic response regulator. VicRK signaling transduction system, 1 of 13 TCS found in *S. mutans*, consists of VicR (a cytoplasmic response regulator) and VicK (a membrane-associated histidine kinase). It was previously demonstrated that VicK and VicR both positively regulated the expression of *gtfB*/*C* by binding their promoter regions. In addition, mutagenesis of the *vicK* and *vicR* coding regions affected sucrose-dependent adhesion and biofilm formation.^26,27^ As a gene in the upstream of *vicRKX* locus, *rnc* can regulate the expression of *vicRKX*. Deletion of *rnc* represses expression of its downstream *vicRKX* gene through mircroRNA-size small RNAs at the posttranscriptional level and subsequently reduces the expression of *gtfB*/*C*/*D*.^28,29^ Cinnamaldehyde, an α, β-unsaturated aromatic aldehyde, had an inhibitory effect on the expression of *vicR*, a part of *vicRKX* TCS in *S. mutans*. As a result, it reduced the expression of *gtfB*/*C*/*D*.^30^ The inhibitory effect on the *vicR* gene is consistent with those observed in botulin (lup-20(29)-ene-3β, 28-diol), a naturally occurring triterpenoid, and botulin (240 μg/ml) reduced the expression of *vicR*, *gtfB*, *gtfC*, and *gtfD* by 2.4-, 8.4-, 8.6-, and 0.2-fold, respectively, compared to the untreated control.^31^ However, the selectivity of these compounds and their mode of action are unknown. RegM, a two-component sensor histidine kinase protein, exhibits high degrees of sequence identity to the catabolite control protein A, a global regulator of sugar metabolism and a primary effector of carbon catabolite repression. RegM could exert its influence on *gtfB*/*C* expression through direct interaction with promoter sequences (51 bp upstream of the start codon).^32^

The sugar phosphotransferase system (PTS), the major sugar uptake system in oral streptococci, especially under carbohydrate-limiting conditions, is involved in the regulation of *gtfs*. The PTS consists of two proteins that are common to all of its substrates, enzyme I (EI) and the heat-stable phosphocarrier protein, as well as sugar-specific permease (EII complexes, consisting of three domains, A, B, C, and sometimes a fourth domain D), which catalyze the transport and concomitant phosphorylation of the substrates.^33,34^ EIIAB^Man^ (encoded by *manL*), as a part of PTS, modulated the expression of *gtfB*/*C* by affecting its promoter.^35^

QS is an intercellular signaling communication network by which bacteria regulate their gene expression in response to environmental cues, such as population density and nutrient availability, by sensing local concentrations of chemical molecules, called autoinducers (AIs).^36^ The most common and widespread QS signal among bacteria is AI-2, which is formed by the enzyme *S*-ribosylhomocysteine lyase (LuxS) that regulates the expression of a large number of genes.^37,38^ As a result, LuxS-based signaling affects *S. mutans* biofilm formation. This is likely due to the altered expression of the *gtfB*/*C* genes in the *luxS* mutant at the mid-log phase.^39^ Recently, it was demonstrated that deletion of *smu_833*, a putative glycosyltransferase, resulted in a decrease in the transcript level of *gtfB*/*C*, but not *gtfD*; furthermore, a point mutation within the catalytic DXD motif of *smu_833* gave rise to a similar inhibitory effect on the expression of *gtfs*, suggesting the importance of the glycosyltransferase enzymatic activity of *smu_833*. However, the exact mechanism by which *smu_833* reduced the expression of *gtfs* is not defined and requires additional research.^40^

Although the expression of *gtfs* is well regulated by its upstream genes, their specific regulation mechanisms are still unknown. Furthermore, the upstream genes may influence the expression of additional genes. Further research is needed before we have the answer. However, targeting upstream signaling pathways indeed reduces biofilm formation of *S. mutans* by affecting the promoter of *gtfs* and subsequent expression of *gtfs*, and it does not change cell survival or the homeostasis of oral flora. Therefore, these upstream signaling genes or molecules may be potential targets, which can be used to develop therapeutic agents that prevent and treat dental caries.

### Mediating the expression of *gtfs* at the transcription level

In addition to targeting upstream signaling pathways or genes, therapeutic agents can directly modulate the expression of *gtfs* at the transcription level, which has been documented widely in *S. mutans*. These therapeutic agents have been mainly found in natural products or their derivatives. For example, citrus lemon oil (CLO), a natural product, has inhibitory effects on *S. mutans* adherence to glass and saliva-coated enamel surfaces through the inhibition of the transcription of g*tfs*.^41^ Apigenin, a 4′,5,7-trihydroxylflavone commonly identified in plant-derived foods and propolis, decreases the expression of *gtfs*, especially *gtf*B and *gtfC* by 50% at a concentration of 0.1 mmol· L^−1^ compared with control, either in solution or absorbed onto sHA beads.^42^ Plant extracts from *Symplocarpus renifolius* significantly downregulate the expression *gtfB*/*C*/*D* (2.0-, 1.5-, and 2.5-fold change, respectively) at 900 μg· mL^−1^, and plant extracts from *Lamium amplexicaule* reduce the expression of *gtfD* (2.0-fold change) at 750 μg· mL^−1^, compared with control.^43^ Deoxynojirimycin (DNJ) from traditional fermented foods significantly reduces the expression of *gtfs* and concurrently decreases the *S. mutans* adherence by 99.97% under the presence of sugar (1% glucose), compared with control.^44^ A similar inhibitory effect of DNJ on the expression of *gtfs* was reported by Hasan et al.^45^ In addition, Hasan et al.^45^ investigated the effect of quercitrin, a flavonoid glycoside, and found that quercitrin suppressed the expression of *gtfs*. More importantly, there was a synergistic effect of anti-cariogenic activity when purified plant-based compounds quercitrin and DNJ are combined, as demonstrated by the significant reduction in the synthesis of both soluble and insoluble polysaccharide.^45^

A great deal of studies has indicated that environmental conditions, such as pH, carbohydrate sources and nutrition availability, and bacterial growth phases mediate the expression of *gtfs*. It is shown that the expression of *gtfB*/*C* increased 30-folds from 48 h growing biofilm growth to 7 days’ growing biofilm when 0.025 mol· L^−1^ sucrose was added in culture, which shows that the expression of *gtfB/C* was significantly different among mature biofilm or relatively young biofilm. This is likely due to the difference in mass transport limitations, the pH, and carbohydrate concentrations experienced by the organisms in various growth environments.^19^ This view was further confirmed in subsequent studies. Environmental pH and carbohydrate source and availability significantly alter the expression of *gtfB*/*C*.^46^ In addition, the expression of *gtfs* was differentially regulated under various growth phases, which is related to cell-density-dependent factors.^47^ Therefore, it is critical to consider the *S. mutans* living environments when evaluating the inhibitory effects of therapeutic agents. The antibacterial mechanisms governing the regulation of the expression of *gtfs* are currently under investigation through different targeted and systems biology approaches, such as transcriptomics analysis. The outcomes from these studies may clarify the underlying inhibitory mechanisms that uncover the molecular links between therapeutic agents and the differential expression of *gtfs*.

## Reducing the Gtfs enzymatic activities

Gene expression studies implicitly showed that changes in mRNA levels have various biological outcomes, presumably mediated by the corresponding changes in protein levels. However, the correlation between gene expression and protein production is not always highly correlated. A variety of posttranscriptional regulatory processes can take place after mRNA is made in controlling steady-state protein abundances, such as mRNA degradation, translational regulation, and protein degradation.^48,49^ To effectively inhibit Gtfs, directly targeting Gtfs activities is a desirable and optimal strategy. A wide range of potential agents has been identified to inhibit Gtfs activities. These various agents inhibited Gtfs activities by different mechanisms, mainly including competitive/noncompetitive/uncompetitive inhibition, and Gtf-based neutralizing antibody approach.

### Competitive/uncompetitive/noncompetitive mechanisms of inhibiting Gtfs

Competitive, uncompetitive, and noncompetitive inhibition are three types of reversible enzyme-inhibitory mechanisms. Inhibitors can prevent a substrate from binding, decrease the enzyme’s catalytic activity, or do both. Competitive inhibitors work by binding at the active site due to their structural similarity to its substrate and prevent the binding of the specific enzyme to substrate. Different from competitive inhibitors, uncompetitive inhibitors have a separate binding site on the enzyme and they do not have an affinity for the free enzyme; instead, they only target the intermediate consisting of the enzyme and substrate complex. Similar to uncompetitive inhibitors, the noncompetitive inhibitor binds to the enzyme and the difference is that they bind to an allosteric site that differs from the active site where the substrate binds.^50^ Classic reversible, noncompetitive inhibitors do not affect substrate binding and vice versa. Thus, noncompetitive inhibitors can equally bind enzyme alone or the enzyme–substrate complex.^50,51^ For better elucidating the inhibitory mechanisms, the inhibitors against Gtfs are broadly grouped into the following categories based on their structures and sources, sucrose and its derivatives, and natural and synthetic products.

The Gtfs have a remarkably high degree of specificity for sucrose and agents that are structurally similar to sucrose are thus becoming a structural inspiration to design and develop Gtfs inhibitors. As structural analogs, sucrose derivatives modified at different positions have been demonstrated to have an inhibitory effect on Gtfs activities by various mechanisms, such as 6-deoxysucrose, 6-thiosucrose, and 4, 6-dideoxysucrose being competitive inhibitors, sucralose, 4-deoxysucrose, and 4-chloro-4-deoxygalactosucrose being noncompetitive inhibitors, and 6, 6’-dithiodisucrose as an uncompetitive inhibitor.^52–54^ Natural products also can inhibit Gtfs activities. For example, Tris, an ethanolamine derivative, was found to act as a competitive inhibitor of GtfB and reduced the activity by 65% at 10 mmol· L^−1^ compared with control.^55^ Green mate (GM) and roasted mate (RM) water extracts, drinks rich in polyphenolic compounds, reduce glucan polymer synthesis in a competitive manner by serving as strong acceptor substrates for Gtfs, of which RM is more effective. At a concentration of 18 mg· mL^−1^, the percent inhibition by the RM extraction was about 25%, whereas by GM it was about 50%.^56^ It has been reported that 7-epiclusianone, isolated from the fruits of *Rheedia gardneriana*, shows 90% inhibition of GtfB and 80% inhibition of GtfC enzymatic activity at a concentration of 100 μg· mL^−1^, compared with control, in a noncompetitive manner and an uncompetitive manner, respectively.^57,58^ In addition, cranberry juice (25%, *v*/*v*) inhibited surface adsorbed GtfB, GtfC, and GtfD activities (70%–80% inhibition of GtfB and GtfC, and 40% inhibition of GtfD compared to control, respectively). It appears that the inhibition of GtfB and GtfC is more effective than the inhibition of GtfD, which could be attributed in part to the content of flavonols, especially quercetin and its glycosides, and polyphenols.^59,60^ Furthermore, quercetin is a noncompetitive inhibitor of Gtfs (40%–70% inhibition at a concentration of 500 μmol·L^−1^ compared with control) and polyphenols, extracted from cacao bean husk or oolong tea leaves, were responsible for inhibiting GtfB (50% inhibition at a concentration of 1.0 μmol·L^−1^ compared with control).^61^ GTI-0163, a mixture of unsaturated fatty acid with a 2:1 ratio of oleic acid to linoleic acid, purified and designated from *Prunus salicina*, shows a 42.4% inhibition rate of Gtfs at 0.05 mmol·L^−1^ in an uncompetitive manner by binding to the enzyme–substrate complex.^62^

Recently, six natural plant products were tested and exhibited considerable levels in inhibiting Gtfs activities. Among them, *Terminalia chebula*, *Psidium guajava*, and *Pongamia pinnata* show uncompetitive inhibition, whereas *Azadirachta indica* displays uncompetitive inhibition, and clove (*Syzygium aromaticum*) and peppermint oil (*Mentha piperita*) have allosteric inhibition (sigmoidal response). Moreover, the polyherbal mouth rinse prepared from all the six plants significantly inhibits Gtfs (95.9%) compared to the chlorohexidine mouthwash’s 54% inhibitory activity.^63^

Although the application of natural products and their derivatives has been documented as one of the most successful approaches for inhibiting activities of Gtfs, some issues need to be resolved, such as the selectivity of these natural compounds is never clear. In this regard, the synthetic products based on the Gtfs structure were designed and developed. #G43, a small-molecule inhibitor, screened in silico against the catalytic domain of Gtfs had a potent affinity for GtfC by the interactions of the ortho primary amide group of the compound with key active site residues of GtfC, and thus inhibiting Gtfs activities and *S. mutans* cariogenicity.^64^ Computer-aided structure-based drug designs guide the development and optimization of lead compounds to increase their affinity, and pharmacodynamic and pharmacokinetic properties.^65^ Strategies employing the in silico methods can be used to predict drug toxicity early on in the drug discovery process, which should facilitate the development of more effective and selective inhibitors.

### Gtf-specific antibodies mediated inhibition

Recognizing the important role of Gtfs in the development of dental biofilms and the pathogenesis of dental caries, developing a vaccine against caries using Gtfs and/or other antigen epitopes as immunogens had been actively pursued and proved to be effective. The approaches include passive immunization involving the use of previously produced antisera elicited by specific Gtf vaccines and active immunization involving the administration of vaccines designed based on targeting immunogenic epitopes of Gtfs.

In passive immunization, ready-made antibodies specific to Gtfs of *S. mutans* are administrated to the host, which should circulate in the blood and provide specific protection against *S. mutans* infection. Antisera are produced using the purified Gtf enzymes, among which antisera to GtfB and GtfC show significant inhibitory effects on both activities regardless of their distribution in solution or adsorbed to a surface, whereas GtfD antisera display more inhibitory effect on GtfD in solution than bound to sHA. One possible explanation for this GtfD unique phenotype is that GtfD in the solution possesses a conformation different from the surface-bound GtfD.^66,67^ Peptides derived from functional motifs within the full-length Gtfs may possess immunogenicity, which has been explored to generate potential vaccines. A self-derived peptide, identical to amino acid sequence 1176–1194 of GtfB, was generated and inhibited GtfB activity in a noncompetitive mode.^68^ To enhance vaccine efficacy, a conjugated antigen containing the two active motifs from different key virulence factors has been constructed and studied. Antibodies against two fusion proteins, consisting of a piece of cell surface protein and GtfB, named PAgA-GB and PAcA-GB, respectively, significantly inhibit the *S. mutans* adhesion to sHA beads in the presence or absence of sucrose. PAgA-GB is composed of the alanine-rich region of the cell surface protein antigen (PAgA) and the GB domain of GtfB, while PAcA-GB is composed of the saliva-binding alanine-rich region of the cell surface protein antigen (PAcA) and GB of GtfB, respectively.^69,70^ The anticaries efficacy of the PAcA-GB antibody was demonstrated in vivo when concentrated immune milk was fed to rats once a day for 55 days.^71^ The inhibitory effects of recombinant protein mentioned above are different in the solution and absorbed onto sHA. However, the sublingual immunization of a recombinant protein consisting of phosphate-binding protein (PstS) has been demonstrated to present dual ability, which not only reduces oral colonization by *S. mutans* but also inhibits the adhesion to abiotic surface administration.^72,73^

However, passive immunization requires repeated administration of large quantities of antibodies; active immunization, therefore, becomes the focus of active research. Local administration of Gtfs antigen in humans after thorough oral prophylaxis retarded the reaccumulation of *S. mutans* due to the induction of salivary immunoglobulin (SIgA).^74^ Among caries vaccines, the new trend is the use of multigenic DNA/recombinant vaccines, which are safe and stable with stronger antigenicity. For example, a recombinant vaccine combining the GB domain (GLU) of *S. mutans* GtfB and thioredoxin of *Escherichia coli* was generated. The vaccine could induce the production of SIgA and reduce colonization and cariogenicity of *S. mutans* in a mice caries model.^75^ Similarly, a fusion anticaries DNA vaccine, pGLUA-P, encoding two antigen fragments, a GLU fragment of GtfB and A-P fragment of PAc, reduced the levels of dental caries induced by *S. mutans* in rats.^76^ To generate a more persistent and larger amount of antigen, a dual promoter consisting of *CMV* and *nirB* promoter driving expression of the genes encoding two virulence factors of *S. mutans*, the saliva-binding region of PAc and the GB region of GtfB. The dual-promoter (*CMV*-*nirB*) vaccine generated a large and persistent amount of antigen protein and was superior to *nirB* promoter used alone in inducing protective immunity against *S. mutans* colonization in BALB/c mice.^77^

In addition, routes of application of vaccines can influence immune responses. Local application to the lower lip area, intranasal, and tonsillar regions showed some promising results. In one study, the application of Gtf vaccine onto the lower lip reduced the indigenous Streptococcal flora compared with a placebo group in young human adults.^74^ Despite these encouraging results, the application of vaccines often requires co-administration of mucosal adjuvants, such as flagellin and enterotoxin from *E. coli*, *Vibrio cholera*, and *Salmonella typhimurium*, which have been successfully used in *S. mutans*, because adjuvants can accelerate, prolong, or enhance immune responses.^78–82^ However, the potential adverse side effects from these adjuvants are unknown.

### Other inhibitory chemicals

Up to now, the inhibitory mechanisms of some effective agents against Gtfs remain to be elucidated. It was reported that CLO, a natural product mixture, effectively reduced the activity of Gtfs (40%–69% inhibition at 18 h, the late-exponential phase) in a dose-dependent manner. Although both limonene and β-pinene, the main components of CLO, have unsaturated bonds in their molecular structure, which may provide binding sites for the side-chain amino acids of Gtfs to diminish its structure stability, future studies are needed to clarify the precise mechanism.^41^ The metal cations and oxidizing compounds have been tested for their ability to inhibit the activities of Gtfs. The results showed that the ability to inhibit Gtfs depends on the Gtfs status, whether they are in solution or adsorbed to the experimental pellicle. In solution, the metal ions including Zn^2+^, Cu^2+^, Fe^2+^, and Fe^3+^, and the oxidizing compounds such as Rose Bengal and hypochlorite significantly inhibit Gtfs activities. In contrast, Gtfs adsorbed to sHA beads exhibit greater resistance to the above inhibitors.^83^ The discrepancy may be due to the conformational changes in the surface attachment resulting in modification of Gtf active sites. Although the exact mechanism by which metal ions inhibit the Gtfs is currently unknown, it has been proposed that metal ions are capable of inactivating Gtf enzymatic activities via covalent modification of amino-acid sulphydryl groups. Reacting with oxygen, these metal ions may oxidatively and covalently modify the Gtfs structure.^83^

## Influences on secretion of Gtfs by dissipating proton motive force across the cell membrane

In *S. mutans*, multiple routes exist for Gtfs secretion, including membrane vesicles, protein secretory (Sec) pathway, and the proton motive force.^84–87^ Among these secretory routes, inhibitors against the proton motive force have been well identified. The proton motive force is required in all bacteria to grow and remain viable under replicating and non-replicating conditions. Metabolic energy is conserved in bacteria by an electrochemical proton gradient across a proton-impermeable membrane.^88^ The electron transport chain components are membrane-bound across the membrane to achieve the import of protons from the cytoplasm and release them to the outside the cell. The proton motive force consists of two gradients: an electrical potential (Δψ, positive_outside_/negative_inside_) and a chemical transmembrane gradient of protons (ΔpH, acidic_outside_/alkaline_inside_).^89–92^ Enzyme secretion by bacterial cells, in general, is coupled to the proton motive force. Some drugs can reduce the proton motive force by enhancing proton permeability and discharging ΔpH across the cell membrane;^93–95^ thus, the side effects and selectivity are not defined.

Fluoride is a well-studied and widely used agent for the prevention of dental caries by inhibiting demineralization and promote remineralization of the teeth and early caries lesions.^96^ Moreover, it has been shown that fluoride has multiple effects on bacteria metabolism and can inhibit bacterial enzymatic activity, which is related to its interference with the transport either directly by dissipating ΔpH or, less directly, by inhibiting the membrane-associated proton-pumping H^+^-ATPase as the organisms attempt to meet the increased demand for proton excretion.^97–99^ It has been demonstrated that fluoride can partially inhibit the secretion of Gtfs by dissipating ΔpH, the proton motive force, across the cell membrane at low concentrations (up to 3.8 p.p.m.) and then modulates the formation of EPS in *S. mutans*.^100,101^

In addition, *tt-*farnesol, a 3,7,11-trimethyl-2,6,10-dodecatrien-1-ol, affects the synthesis of EPSs by diminishing the proton motive force across the cell membrane and reducing the secretion of Gtfs.^102^ These agents exert inhibitory effects through impairing secretion function, not decreasing the expression of Gtfs. Therefore, when combined with additional cariostatic agents inhibiting the expression of Gtfs, it synergistically enhances their effectiveness. The fact is that topical application of apigenin and tt-farnesol (1.33 mmol·L^–1^ each), both identified in propolis, in combination diminishes the incidence of smooth-surface caries (up to 60% reduction) and displays lower smooth-surface caries severity than either compound alone.^103^ Similar enhanced effectiveness has been demonstrated in the combination of fluoride and other agents. The combination of apigenin, tt-farnesol, and fluoride was the more effective treatment in reducing the biomass and insoluble glucans of *S. mutans* biofilms than apigenin and fluoride, and tt-farnesol and fluoride through different mechanisms: apigenin inhibiting the expression of Gtfs, and tt-farnesol and fluoride both affecting the secretion of Gtfs.^102^

## Conclusions and future perspectives

Recognizing the crucial role of Gtfs in the development of dental biofilms and the pathogenesis of dental caries, interference with Gtfs sparked remarkable interests among alternatives for drug development. Importantly, this approach is more advantageous in reducing the expression of the virulence factors of *S. mutans* without suppressing the resident oral flora. We have broadly categorized these agents according to their inhibitory mechanisms: modulating the expression of *gtf* genes, affecting the Gtfs activities, and reducing the secretion of Gtfs.

Among these Gtfs inhibitors, natural products have long been recognized as an important source of therapeutic medicines. Current challenges and difficulties in the application of natural products include lack of standard procedures, isolation of pure chemical products or compounds, and elucidation of modes of action, owing to their complex chemistry and variable composition, as well as unknown interactions between compounds and Gtfs active sites. Apart from natural compounds, synthetic products, antibodies, and metal ions have also been recognized in inhibiting Gtfs activities; however, their inhibitory mechanisms are not known and still under investigation. The convergence of new analytical technologies with advances in synthetic drug-like small-molecule compounds targeting Gtfs using computer-aided designs and innovative isolation and preparation methodologies has opened the door to a new era in the development of novel anticaries therapies and the understanding regulatory mechanisms.^104–111^ The combinational therapeutics of different inhibitors including multigenic DNA/recombinant vaccines have shown significantly improved anti-cariogenic efficacy, a promising strategy to design and develop anticaries agents.

In summary, Gtfs are the crucial virulence factors in the etiology and pathogenesis of dental caries, which provides a key target to the discovery of effective inhibitors that affect gene expression or/and enzyme activity, or secretion. Inhibiting Gtfs led to the reduction in *S. mutans* adhesion and accumulation on the tooth surfaces, and hence the formation of dental biofilms. However, different therapeutic strategies display diverse selectivity and sensitivity by different modes of action. Therefore, understanding new underlying molecular mechanisms of inhibiting Gtfs is critical for developing anticaries agents that should guide the combination of agents targeting additional virulence factors.
